# Integrating Population Genetics With Long‐Term Environmental Monitoring to Evaluate and Guide Vernal Pool Creation for Amphibian Conservation

**DOI:** 10.1002/ece3.70431

**Published:** 2024-10-19

**Authors:** Declan M. Winters, Emily Wilson, Stephanie S. Coster, Megan B. Rothenberger

**Affiliations:** ^1^ Department of Biology Lafayette College Easton Pennsylvania USA; ^2^ Department of Microbiology, Immunology, and Molecular Genetics (MIMG) University of California Los Angeles Los Angeles California USA; ^3^ Institute for Quantitative and Computational Biosciences (QCBio) University of California Los Angeles Los Angeles California USA; ^4^ Department of Human Genetics, David Geffen School of Medicine University of California Los Angeles Los Angeles California USA; ^5^ Environmental Science and Studies Program Lafayette College Easton Pennsylvania USA; ^6^ Department of Earth and Environment Boston University Boston Massachusetts USA; ^7^ Department of Biology Randolph‐Macon College Ashland Virginia USA

**Keywords:** amphibian, conservation, habitat creation, long‐term monitoring, population genetics, vernal pool

## Abstract

The decline of biodiversity, particularly among amphibians, is strongly associated with habitat loss and fragmentation. Vernal pools are a critical ecosystem for many pool‐breeding amphibians, but they are often overlooked in wetland protection guidelines. Mitigation efforts include vernal pool creation and restoration, but these efforts have varying success in replacing lost functions. This study investigates the success of created vernal pools through long‐term environmental monitoring of wood frogs and spotted salamanders (2014–2023) and integrates population genetics to assess the local population health of the wood frog. First, we monitored and compared environmental parameters and reproductive success of indicator species between natural and created pools in a Pennsylvania state park. We then used microsatellite loci to assess within‐ and between‐pool measures of genetic diversity, population structuring, and gene flow for wood frogs. We found two carefully designed created pools positively contributed to local amphibian population persistence by maintaining similar measures of genetic diversity as compared to natural pools. On the other hand, one poorly created pool was genetically distinct and acted as a population sink. Although our findings offer valuable insights, they are based on a limited sample and may not fully represent the broader landscape. However, by integrating genetic information into long‐term monitoring datasets, our interdisciplinary approach enhances our understanding of amphibian population dynamics in vernal pool ecosystems. Our findings imply that the most important factors for restoration practitioners to consider when creating or restoring vernal pools are hydroperiod (12–35 weeks), volume (> 50 m^3^), depth (≥ 30 cm), and surrounding forest land cover (> 60%). These variables are better predictors of indicator species success than pool type (i.e., natural or created). Ultimately, this study emphasizes the need to accompany restoration efforts with long‐term monitoring programs that can be used to make adaptive management decisions in an era of extreme environmental change.

## Graphical abstract

This study investigates the success of created vernal pools in sustaining local amphibian populations (i.e., wood frogs and spotted salamanders) in a Pennsylvania state park from 2014 to 2023. By comparing environmental parameters and genetic diversity between natural and created pools, we found that well‐designed created pools positively contributed to wood frog population persistence, while one poorly designed pool acted as a population sink. Our findings underscore the importance of considering key environmental factors such as hydroperiod, volume, depth, and surrounding forest cover in vernal pool restoration while also integrating these data with population genetics to guide adaptive management strategies in the face of environmental change.

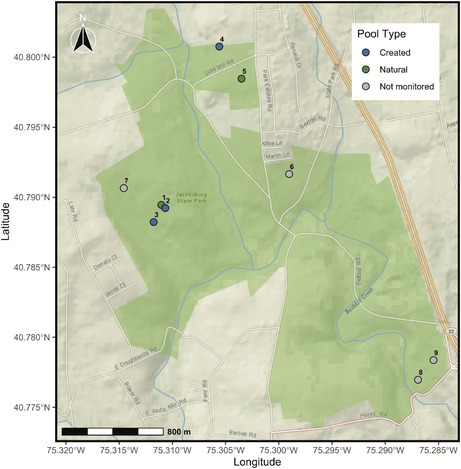

## 1 Introduction

The primary cause of biodiversity declines at the level of ecosystems, species, and genetic variation is habitat loss (Brooks et al. 2002; Díaz et al. 2019). Habitat loss does not always equate to complete destruction. Habitat fragmentation and degradation may also mean the habitat is effectively “lost” to species that cannot tolerate these changes. Amphibians are known to be more vulnerable than other vertebrate animals to habitat loss and fragmentation because of their biphasic life history and limited dispersal ability (Lichko and Calhoun 2003; Tan, Herrel, and Rödder 2023). In northeastern North America, 56% of amphibian species use vernal pools to breed, develop, forage, and hibernate (Calhoun and deMaynadier 2008). Vernal pools, which are temporary depressional wetlands, are often excluded from wetland protection guidelines due to their small size and temporary hydrology (Semlitsch and Bodie 1998; Calhoun et al. 2017). Despite the rich biodiversity vernal pools support, current federal wetland regulations under the Clean Water Act in the United States only apply to large wetlands (i.e., > 0.04 ha) that have permanent surface connections to navigable waters (Mushet et al. 2015). As a result, amphibian presence, abundance, and species diversity in northeastern North America decline as vernal pools and upland habitat are lost or embedded in a matrix of roads and houses (Windmiller and Calhoun 2008).

Continued loss and degradation of vernal pools has led to mitigation efforts that include vernal pool creation (i.e., construction of a new pool where it did not formerly exist) and restoration (i.e., rehabilitation of existing vernal pools) (Calhoun et al. 2014; Schlatter, Faist, and Collinge 2016). However, creation and restoration of vernal pools is notoriously difficult because of their seasonal water regime and the need to protect adjacent intact, largely forested post‐breeding habitat for biphasic amphibians (Lichko and Calhoun 2003; Semlitsch and Skelley 2008; Calhoun et al. 2017). Conserving and restoring landscapes that contain multiple vernal pools with different hydroperiods connected by high‐quality upland forest habitat enables populations to adapt to a changing environment (Calhoun et al. 2017). Habitat connectivity is especially important to the regional viability of vernal pool‐breeding amphibians, such the spotted salamander (*Ambystoma maculatum*) and the North American wood frog (*Lithobates sylvaticus*), which require upland habitat to forage and hibernate but return to natal ponds for breeding (Berven and Grudzien 1990). Wood frogs are highly philopatric with well‐developed homing abilities. Berven and Grudzien (1990) reported that about 18% of metamorphosing froglets recaptured at maturity dispersed to neighboring pools to breed whereas 100% of adults were faithful to the pools in which they first bred. This site fidelity, coupled with their reliance on both aquatic and terrestrial habitats, closely links their survival and reproductive success to the health of these habitats. Consequently, they are excellent bioindicators for assessing the effectiveness of vernal pool restoration efforts.

Although conservationists have emphasized that created pools often do not replace the functions lost in the destruction of natural pools (De Weese 1998; Calhoun et al. 2014), more vernal pool restoration studies are indicating that well‐designed created pools with high‐quality post‐breeding habitat can be beneficial to amphibian populations in the long term. Regardless of the type of vernal pool (i.e., natural, created, or restored), reproductive success of amphibians appears to be associated with time since vernal pool creation, pool size, hydroperiod, forest cover within 1000 m of a pool, and predator abundance (e.g., Vasconcelos and Calhoun 2006; Skidds et al. 2007; Millikin et al. 2019, 2023; Rothenberger et al. 2019; Rothenberger and Baranovic 2020). Restoration practitioners aiming to create vernal pools should carefully consider these factors during the planning and implementation phases of a project.

Achieving desired outcomes also requires that creation or restoration attempts be accompanied by long‐term monitoring and evaluation (Schlatter, Faist, and Collinge 2016; Calhoun et al. 2017). Most regulatory agencies require 3–5 years of monitoring following creation or restoration of vernal pools, but short‐term monitoring results may not be indicative of long‐term success (Schlatter, Faist, and Collinge 2016). Post‐restoration monitoring should include factors known to alter reproductive success of vernal pool indicator species such as vernal pool hydroperiod and continue for at least 10 years to properly account for soil compaction during construction, vegetation growth, and establishment of predators and prey (Calhoun et al. 2017; Sueltenfuss and Cooper 2019; Rothenberger and Baranovic 2020).

There are numerous tools that restoration practitioners can use to evaluate vernal pool “success.” Amphibian egg mass counts are one of the most common measures of success, but some vernal pools may function as population sinks where individuals breed but larvae are unable to survive to metamorphosis (Calhoun et al. 2014; Rothenberger and Baranovic 2020). Therefore, larval survival (i.e., proportion of eggs that metamorphosize) is considered a superior metric to egg mass counts (Calhoun et al. 2014). Understanding population genetics can help assess success by exploring genetic diversity and connectivity among natural, restored, and created vernal pools (Furman et al. 2016; Neal et al. 2020; Skibbe et al. 2021; Millikin et al. 2023). High levels of genetic diversity of vernal pool indicator species, as indicated by allelic richness and heterozygosity, would suggest greater gene flow and connectivity, low inbreeding, enhanced reproductive fitness, and reduced risk of extinction (Reed and Frankham 2003). Low genetic diversity may indicate either founder or bottleneck events that can lead to higher levels of genetic drift where a small population size results in the loss of rare alleles (Nei, Maruyama, and Chakraborty 1975).

The first objective of this study was to explore the success of vernal pool creation through long‐term monitoring (i.e., 2014–2023). In order to isolate factors that are most important for native species success, our monitoring study included three created and two natural pools within a Pennsylvania state park. We compared habitat variables and larval survival of the wood frog and spotted salamander, both obligate vernal pool‐breeding amphibians, among pool types and evaluated correlations between habitat variables and reproductive success.

The second objective was to incorporate population genetics tools to assess population establishment and habitat connectivity for the wood frog specifically, because it is the most abundant obligate vernal pool species at the study site. Ecological restoration and management of vernal pools should seek to increase genetic diversity by maximizing gene flow through dispersal (McKee et al. 2017). The fact that the three created and two natural pools included in our study are distributed in two locations with different degrees of habitat fragmentation by roads enabled us to use wood frog genetic differentiation among pools to assess connectivity and gene flow. Previous research has shown that roads can significantly impact genetic structure between wood frog breeding sites (Crosby, Licht, and Fu 2009; Gabrielsen et al. 2013). Although the road in our study is smaller, fieldwork at this site showed that wood frog movement patterns were affected by the road, potentially hindering their dispersal ability (Engberg and Rothenberger 2015). We wanted to explore how created pools contribute to gene flow within a fragmented context and whether there were signs of a bottleneck or founder effect in the created pools. While other studies on vernal pool amphibian population genetics in created pools exist (Furman et al. 2016; Neal et al. 2020; Skibbe et al. 2021; Millikin et al. 2023), ours is unique in that it integrates genetic information with a long‐term vernal pool monitoring dataset. This long‐term vernal pool dataset is rare and valuable because it enables us to isolate factors that are most important for amphibian reproductive success.

## 2 Materials and Methods

### 2.1 Study Area

**FIGURE 1:**
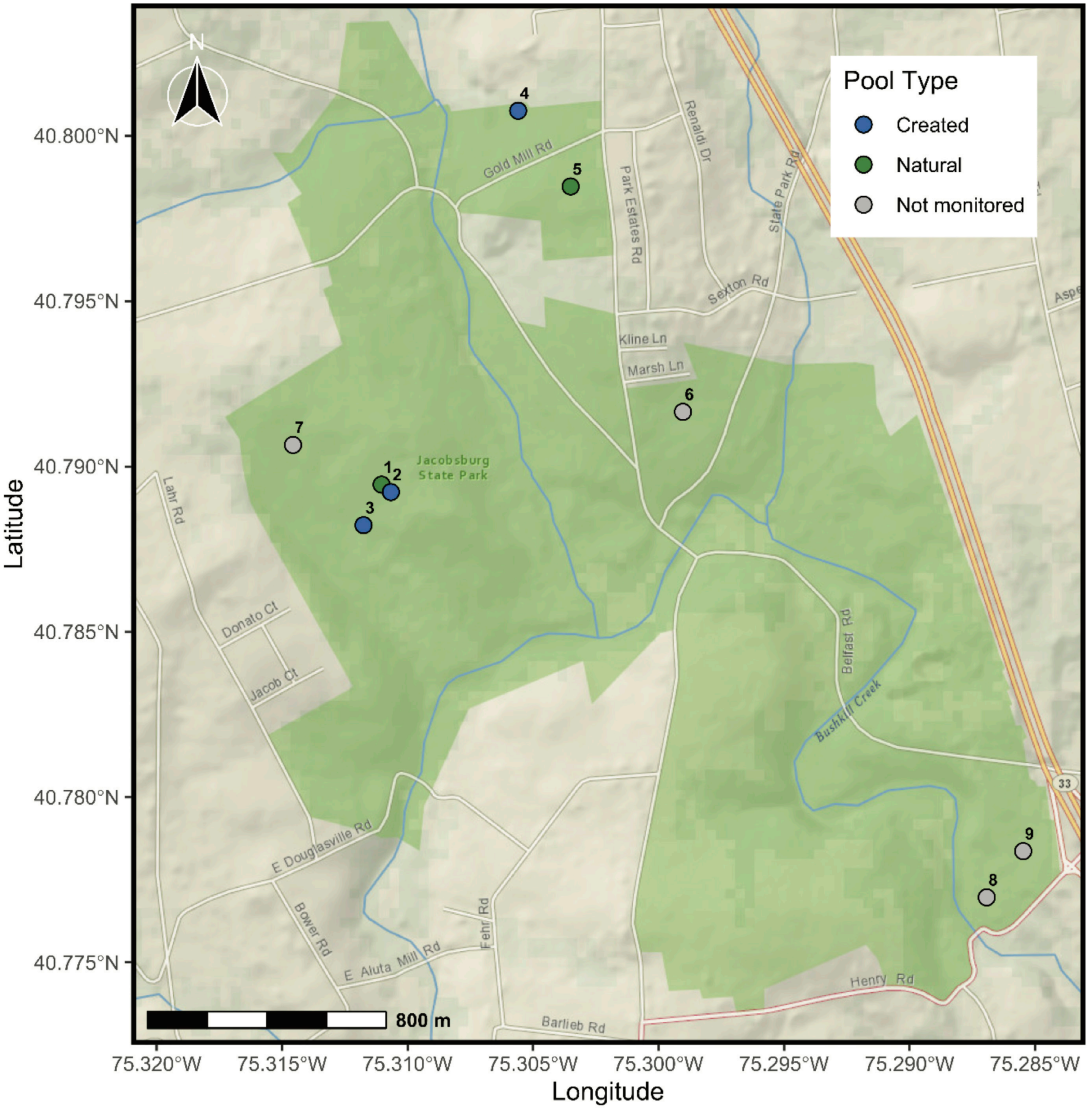
Map of the study area, which included two natural and three created pools situated in two locations within Jacobsburg State Park in eastern Pennsylvania.

Jacobsburg State Park is a 4.73 km^2^ state park approximately 11 km northwest of Easton, PA in Northampton County. Included in this study are two natural and three created pools situated in two locations within the park (Figure 1). While the park manager had information on the age of the created pools and knew they were constructed by youth organizations and college students under the guidance of a former park manager, no formal documentation of restoration objectives, design criteria, or initial monitoring data exist. The created pools were constructed near existing natural pools but in areas of the park where vernal pools did not formerly exist. A natural pool (JSP1) and two unlined pools created in 2011 and 2008, respectively (JSP2 and JSP3), are situated in an isolated location, which is more than 1000 m from the closest vehicle‐accessible road. These three pools are within 125 m of one another with no obstacles between them. The second location within Jacobsburg State Park is roughly 800 m away from the first and is near a residential community. This site includes a synthetically lined pool created in 2008 (JSP4) and a natural pool (JSP5) positioned approximately 250 m from one another and less than 100 m from two asphalted roads. At this location, one road bisects JSP4 and JSP5 (Figure 1). Although the exact number and locations of all vernal pools within the park remain unknown, Figure 1 shows the GPS coordinates of 4 additional pools that were excluded from routine monitoring due to resource constraints and a lack of information regarding pool type (i.e., natural, created, or restored).

### 2.2 Vernal Pool Monitoring and Habitat Characteristics

Methods for monitoring hydrology, water chemistry, and wood frog and spotted salamander egg mass density and larval survival are described in Rothenberger et al. (2019). Environmental monitoring for these parameters began in 2014 and is ongoing. Each year, we collected data on hydrology, water chemistry, and egg mass abundance on a weekly basis from the first week of March (i.e., approximately one week before typical initiation of oviposition in this area) until all eggs have hatched and decomposed. We estimated larval abundance immediately after hatching and during metamorphosis in all pools in 2018, 2019, and 2020. Finally, we estimated overall survival for each species as a proportion by dividing the number of metamorphosing individuals by the number of eggs in each pool. Because egg masses, but not individual eggs were counted, we calculated overall survival indirectly using a mean clutch size of 500 eggs per clutch for wood frogs and 100 eggs per clutch for spotted salamanders (Seigel 1983; Berven 1990). Methods for larval sampling were approved by the Institutional Animal Care and Use Committee (IACUC) at Lafayette College.

We measured canopy coverage during the growing season using a convex spherical crown densiometer. Readings were taken in the center of each pool and at distances of 10, 65, and 120 m in each of the cardinal directions leading away from the pool. At each of those locations, four densiometer readings were taken in the cardinal directions surrounding that location. We estimated overstory density for a particular pool by averaging these 13 readings (i.e., 1 pool center +4 cardinal directions × 3 distances). We used Geographic Information Systems (GIS) to classify and compare land use surrounding each of the 5 vernal pools. Land Use Land Cover (LULC) data for this analysis were taken from the United States Geological Survey (USGS) 2011 National Land Cover Database which has a spatial resolution of 30 m. The LULC grid was imported into the GIS interface and reclassified using the Reclassify tool available in Spatial Analyst to include just five general categories: (1) developed, (2) forested, (3) wetland, (4) agriculture, and (5) water. We used the Spatial Analyst “tabulate area” function to calculate the area of each land use category within polygons with a radial distance of 1000 m of each pool. This size was chosen because studies have shown that juvenile wood frogs can disperse, on average, as far as 1000 m from their birth site. This area includes the three habitat types, or life zone, utilized by vernal pool‐breeding amphibians: the pool for breeding, forested wetlands or hillside seeps for summer refugia, and well‐drained upland forests for hibernation (Baldwin, Calhoun, and deMaynadier 2006). We then compared the proportion of each land cover type within the polygon among the natural and created pools.

We used one‐way analysis of variance (ANOVA) and Tukey's multiple comparison test to compare variables among sites (i.e., isolated [*n* = 3] and fragmented [*n* = 2]) and pool types (i.e., natural pools [*n* = 2] and created pools [*n* = 3]). A separate model was run for each dependent variable, and pool was included as a random effect for the egg mass and water chemistry models to account for repeated samples within the same pools for these parameters.

Given that the monitoring portion of this study resulted in a high‐dimensional dataset, we used nonmetric multidimensional scaling (NMDS), considered the most effective ordination method for ecological data (McCune and Grace 2002), to investigate potential environmental predictors of amphibian success in vernal pools. All NMDS ordinations were carried out using the “metaMDS” function in the VEGAN v.2.6.4 R package (Oksanen et al. 2022; R Core Team 2022). Biotic parameters (egg mass abundance and density and overall survival for the wood frog and spotted salamander) were calculated, and the Bray‐Curtis index was used as a measure of dissimilarity among data for each pool in each year. We included year, pool type, and hydroperiod class in the analyses as categorical variables. Hydroperiod categories were roughly based upon the hydrologic classification for vernal pools proposed by Colburn (2004) and included highly ephemeral (mean hydroperiod of < 12 weeks), short‐cycle (12–20 weeks), long‐cycle (20–35 weeks), and semipermanent pools (> 35 weeks). We then fit our biotic factors as vectors to the NMDS axes using the “envfit” function in VEGAN to visualize their strength and direction. To better understand possible contributions of environmental variables to indicator species success, we fit vectors of overhead canopy coverage, proportion of forested, agricultural, and developed land within a radial distance of 1000 m of each pool, and within‐pool water quality (conductivity and % DO saturation) to the same NMDS axes with the “envfit” function.

### 2.3 Sample Collection and Genotyping

**FIGURE A1:**
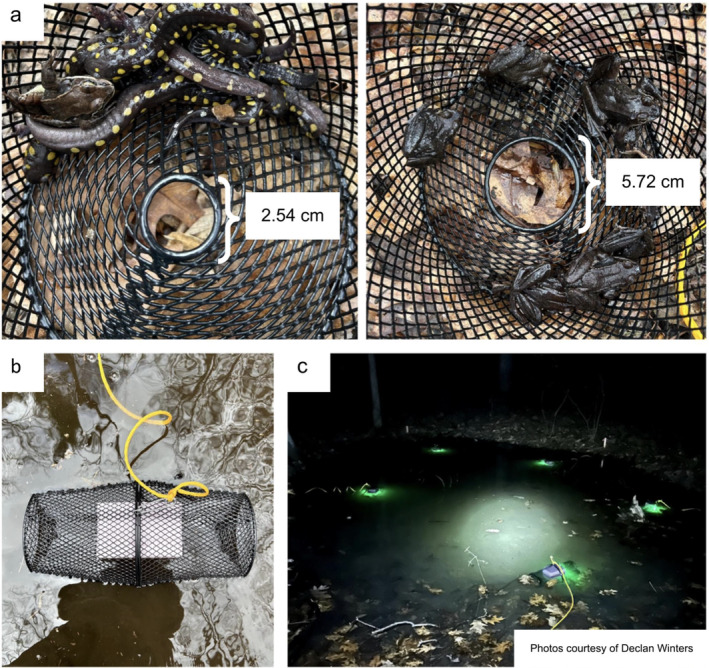
Methods used to collect adult wood frogs with aquatic funnel traps. (a) Internal view of the aquatic funnel traps' openings with adult wood frogs and spotted salamanders captured in two aquatic funnel trap variants. (b) A deployed aquatic funnel trap baited with a glow stick and foam float to partially raise the unit above the surface of the pool. (c) Funnel traps with openings positioned perpendicular to the bank every ~10 m along the perimeter of JSP2 with glow sticks illuminating at night.

Genetic material was collected from wood frogs in March 2022 and 2023 using two different methods. In 2022, we collected one egg from ~20 wood frog egg masses at each pool. When possible, eggs were selected from egg masses in different areas of the pools. In 2023, we attempted to increase sample sizes by performing a toe‐clipping procedure to obtain a tissue sample. We collected adult wood frogs in aquatic funnel traps (Frabill, Plano Molding Company, Plano, Illinois, USA) illuminated with green glow sticks (Appendix 1 Figure A1) (Antonishak, Muñoz, and Miller 2017). Details of sample collection are provided in Appendix 1. Methods for genetic material collection were approved by the Lafayette College IACUC and the Department of Conservation and Natural Resources (DCNR) Bureau of State Parks.

Tissue samples were stored in 95% ethanol at −20°C prior to DNA extraction using QIAGEN DNeasy Blood & Tissue kits. Nine DNA primer pairs designed to amplify tetranucleotide microsatellites in the wood frog were used (Julian and King 2003). We used additional universal fluorescent‐dye‐labeled (6‐FAM, HEX, NED) primer tails in the assay to label the microsatellite loci following Blacket et al. (2012). A polymerase chain reaction (PCR) multiplex design was used to divide loci between three multiplexes: Multiplex A (C52, C83, D32, D55), Multiplex B (C11, C41, C63), and Multiplex C (D20, D77) (Appendix 2 Table A1). Concentrations of primers varied (Appendix 2 Table A2). We used hot start PCR with the QIAGEN Type‐it Microsatellite PCR Kit. Cycling conditions involved an initial activation step at 95°C for 15 mins followed by 28 cycles with a denaturation at 94°C for 30 s, annealing at 58°C for 90 s, extension at 72°C for 60 s, and a final extension step at 60°C for 30 min. All reactions were visualized on 2% agarose gels with a current of 100 V for 1 h to check for successful amplification of fragments, and we re‐amplified problem samples.

Fragment analysis was performed using capillary electrophoresis on an Applied Biosystems Capillary 3730xL DNA Analyzer using 500 ROX as a size standard. In each run, we included a positive and negative control. Geneious Prime v2023.2 (Biomatters Ltd.) was then used to manually fit ladders, call peaks, predict bins, and display alleles. We dropped all samples that failed to amplify in > 3 loci.

### 2.4 Analysis of Within‐Population Genetic Diversity

To determine whether we could combine samples that were collected in two consecutive years (JSP1, JSP2, JSP3, JSP5), we tested the annual variation of allele frequencies between samples from both years using the exact G test in GENEPOP on the web v4.7.5 (Raymond and Rousset 1995; Rousset 2008) with Bonferroni adjustment (ɑ = 0.05).

We checked for scoring errors and large‐allele dropout using MICRO‐CHECKER v2.2.3 (Van Oosterhout et al. 2004). We calculated null allele frequencies using the Brookfield 1 estimator (Brookfield 1996) in GENEPOP (Raymond and Rousset 1995; Rousset 2008). Loci with null allele frequencies > 20% were dropped from all downstream analyses (Dakin and Avise 2004). After dropping null alleles, we used POWSIM v4.1 (Ryman and Palm 2006) to estimate whether the number of remaining loci and their allelic frequencies provided enough power to detect significant genetic differentiation. For each test, we used the sample sizes and the number of alleles and their frequencies from our data set. We executed 1000 independent simulations of populations with an effective population size (*N*_*e*_) from 50 to 2000 undergoing drift over a period of 2–25 generations. *N*_*e*_ values were chosen based on values suggested for ranid frog populations (Hoffman, Schueler, and Blouin 2004). The proportion of simulations identified as significantly differentiated using both chi‐square and the Fisher's exact tests (*p* < 0.05) provides a measure of the power of our microsatellites to identify population differentiation at the average *F*_*ST*_ for each drift scenario.

Microsatellite genotypes were used to perform sibship reconstruction in COLONY v2.0.6.8 (Jones and Wang 2010). In COLONY, we set male and female mating to polygamous without inbreeding. We conducted a medium run with full likelihood and omitted a sibship prior. We ran the analysis in duplicate and subsequently haphazardly removed all but one individual from any full‐sibling family with a sibship probability greater than 0.9 in both runs. Removing siblings not only minimizes the degree of family structure present in our sample set but also improves congruence in sampling design between populations sampled both at the egg stage and as free‐swimming adults when unintentionally collecting siblings is more likely (Seigel 1983).

We calculated departures from Hardy–Weinberg equilibrium (HWE) and linkage disequilibrium (LD) using Fisher's exact test approximations in GENEPOP (Raymond and Rousset 1995; Rousset 2008) with a Bonferroni‐corrected *p*‐value (ɑ = 0.05) for multiple tests (Markov chain parameters: 10,000 dememorization steps, 1000 batches, and 10,000 iterations per batch).

We assessed genetic diversity by calculating the average number of alleles across loci (*A*_*O*_) in GenAlEx v6.51b (Peakall and Smouse 2006, 2012) and rarefied estimates of both allelic richness (*AR*) and private allelic richness (*N*_*p*_) in the program HP‐RARE v1.0 (Kalinowski 2005). Rarefaction is a statistical procedure that, in this case, estimates a value of allelic richness based on the smallest number of samples taken since increasing sampling effort often yields increased richness (Grey et al. 2018). We calculated observed heterozygosity (*H*_*O*_), expected heterozygosity (*H*_*E*_), and Wright's inbreeding coefficient (*F*_*IS*_) using GenAlEx (Peakall and Smouse 2006, 2012). In COLONY (Jones and Wang 2010), we calculated the effective number of breeders (*N*_*b*_) at each vernal pool using the aforementioned program parameters. This analysis was performed alongside the identification of full‐sibling families because accurate sibship reconstruction in COLONY provides information on relatedness and variance in family size that is used to estimate *N*_*b*_ (Ackerman et al. 2017).

### 2.5 Analysis of Population Differentiation and Structure

To quantify the degree of genetic differentiation among and within populations, we calculated pairwise *F*_*ST*_ values in ARLEQUIN v3.5.2.2 (Excoffier and Lischer 2010) and statistical significance of pairwise values based on a permutation test (Manly 2018) with 1000 iterations. The sequential Bonferroni procedure was applied over population pairs in determining significance levels.

We used the program STRUCTURE v2.3.4 (Pritchard, Stephens, and Donnelly 2000) as a second test to delineate populations. Since wood frogs are highly philopatric (Berven and Grudzien 1990), studies evaluating this species with STRUCTURE commonly assume each collection site (i.e., vernal pool) to be representative of a single, randomly mating population of wood frogs. This includes a fine‐scale analysis (pools < 1 km apart) as is the case with our study (e.g., Newman and Squire 2001; Squire and Newman 2002; Crosby, Licht, and Fu 2009; Gabrielsen et al. 2013; Peterman et al. 2013). STRUCTURE uses a Bayseian clustering approach to determine the number of distinct genetic groups (*K*) given similarities between the genotypic data of all sampled individuals using an admixture model (Pritchard, Stephens, and Donnelly 2000). We set the range of *K* from 1 to 5 with 10 runs at each *K*‐level and correlated allele frequencies at a burn‐in length of 200 k Markov chain Monte Carlo (MCMC) iterations followed by 500 k iterations with consideration of prior location. These parameters were chosen based on similar studies (Richardson 2012; Peterman et al. 2013; Skibbe et al. 2021). POPHELPER v1.0.10 (Francis 2017) was used to determine the optimal number of genetic clusters by estimating the average log likelihood of the data (LnPr(*X*|*K*)) and *ΔK* (Pritchard, Stephens, and Donnelly 2000; Evanno, Regnaut, and Goudet 2005). Higher values of these metrics indicate stronger support for a given number of genetic clusters. Using multiple runs to evaluate *K* in STRUCTURE can produce unique solutions due to label switching across replicates (Jakobsson and Rosenberg 2007). Therefore, post‐processing of this model‐based population structure method was performed with CLUMPAK v1.1 (Kopelman et al. 2015) to produce the final output.

Following the methods of Furman et al. 2016, we tested three different Analysis of Molecular Variance (AMOVA) models using GenAlEx (Peakall and Smouse 2006, 2012) to determine if populations at natural and created vernal pools can be differentiated. The first model tested if the three pools at the isolated site were genetically unique from the other site with two pools within the park. The second model tested if natural pools were different from created pools by grouping all natural pools together and all created pools together. The third model was similar to the second but instead assigned each of three created pools into its own group and all natural pools into one group (4 groups total). This statistical method partitions variance into various hierarchical levels and looks for where variation can be explained. In each model, we tested 4 levels of hierarchical variation. These levels included among groups, among pools within groups, among individuals within pools, and within individuals. We ran all AMOVA analyses with 9999 permutations to test for significance and used locus‐by‐locus analyses.

### 2.6 Gene Flow

We used the genetic assignment method in BAYESASS v3.0.4 (Wilson and Rannala 2003) to estimate recent migration rates over the last several generations among the natural and created pools. This analysis has been used to evaluate close proximity vernal pool‐breeding populations (e.g., Scherer et al. 2012 for wood frogs; Wang and Schaffer 2017 for *Ambystoma californiense*; Martínez‐Gil et al. 2023 for *Hyla molleri* and *Pelobates cultripes*). BAYESASS uses a fully Bayesian MCMC resampling method to estimate asymmetrical rates of gene flow between populations and calculate confidence intervals for results that would indicate uninformative data such as those that do not contain sufficient variation to estimate dispersal with high confidence (Wilson and Rannala 2003, Pearse and Crandall 2004). We performed a run with 10 M MCMC iterations, discarding 1 M as burn‐in, and sampling the chain every 100 generations. We adjusted the parameters for migration rate, allele frequency, and inbreeding coefficient to 0.50, 0.60, and 0.75, respectively, to ensure that the acceptance rate fell between acceptable values of 20% and 60% (Rannala 2015).

### 2.7 Bottleneck Evaluation

We used three methods to detect molecular signatures of bottlenecks. The first method in the program BOTTLENECK v1.2.02 (Cornuet and Luikart 1996; Piry, Luikart, and Cornuet 1999) examines genotypes for significant heterozygote excess and deficiency with a nonparametric two‐tailed Wilcoxon test, the most appropriate and powerful test for datasets with less than 20 loci (Piry, Luikart, and Cornuet 1999). We used 5000 replicates and two models of microsatellite mutation: the Two Phase Model (TPM) and the Stepwise Mutation Model (SMM). Microsatellites are generally believed to mutate according to the TPM (Di Rienzo et al. 1994), but this model can add unnecessary parameters and can lead to poor estimates (Sainodiin et al. 2004). The SMM, on the other hand, offers the most conservative test for bottlenecks (Cornuet and Luikart 1996).

Second, in BOTTLENECK, allelic frequency distributions were assessed using the mode‐shift indicator (Luikart 1998). The presence of an L‐shaped frequency indicates a healthy population with a high proportion of low‐frequency alleles present. Population bottlenecks cause a characteristic mode‐shift distortion in the distribution of allele frequencies. Bottlenecks cause rare alleles at low frequency (< 0.1) to become less abundant than alleles in one or more intermediate allele frequency class (e.g., 0.1–0.2).

In ARLEQUIN (Excoffier and Lischer 2010), we also calculated *M*, a ratio based on the number of alleles to the allele size range to detect the signature of a past genetic bottleneck (Garza and Williamson 2001). This measure is based on the assumption that in a bottleneck event, the number of alleles decreases faster than the allelic range because the latter is only reduced if the shortest and/or longest allele is lost, whereas the loss of any allele reduces the former (Garza and Williamson 2001). We considered *M* < 0.68 to be indicative of a bottleneck and *M* > 0.80 suggestive of no reduction in effective population size (Garza and Williamson 2001).

## 3 Results

### 3.1 Monitoring Trends

**FIGURE 2:**
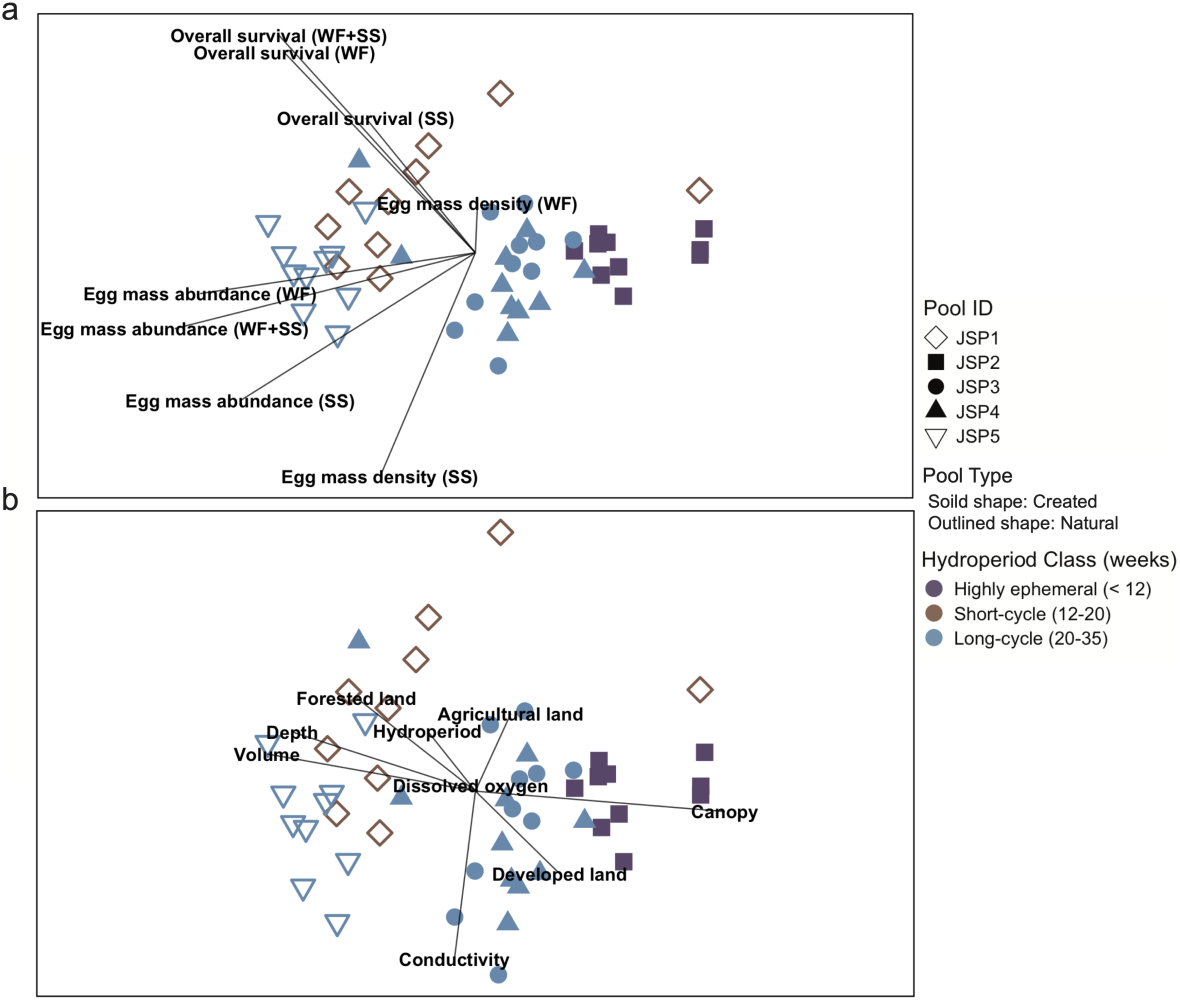
Ordination of vernal pools in each year sampled (2014–2023) by measures of indicator species success. Each point represents one pool in a particular year, and points are shaped by pool ID and colored by hydroperiod class. Outlined points represent natural pools, and solid points represent created pools. Distances between points suggest relative similarities in reproductive success of indicator species (stress value = 0.11). (a) Vectors indicate the strength and direction of biotic parameters (WF = wood frog, SS = spotted salamander). (b) Vectors indicate the strength and direction of environmental parameters.

As indicated by the clear separation of natural and created pools in the ordination diagrams (Figure 2), NMDS of vernal pools by indicator species success indicates that egg mass abundance and overall survival of juveniles is generally higher in natural pools (outlined shape) than created pools (solid shapes; Figure 2a). As indicated by the length of the vectors representing environmental parameters, pools with greater amphibian success were larger, deeper, and surrounded by higher proportions of forest land cover within the surrounding 1000 m (Figure 2b). Pools with short‐ to long‐cycle hydroperiods (i.e., 12–35 weeks) supported better overall survival than the highly ephemeral pool (Figure 2). Canopy cover directly over pools was > 90% for all the pools, but tree canopy was slightly more open at the larger, more successful natural pools (Figure 2b). Mean egg mass abundance was 53.1 ± 8.7 (range = 0–250) for wood frogs and 8.6 ± 2.4 (range = 0–81) for spotted salamanders. Mean larval survival was 4.3% ± 1.2% (range = 0%–44.2%) for wood frogs and 2.0 ± 0.9 (range = 0%–37.2%) for spotted salamanders. For wood frogs, mean egg mass abundance (91.3 ± 17.5) and larval survival (9.0 ± 2.5) at natural pools were significantly higher than egg mass abundance (27.6 ± 4.9) and larval survival (1.2 ± 0.6) at created pools. The smallest created pool, JSP2, never supported spotted salamanders and was the only pool that had no wood frogs survive through metamorphosis during the study period (Table 1).

**TABLE 1:** Summary of vernal pool characteristics and wood frog success.

Pool	Pool type	Depth (cm)	Volume (m^3^)	Hydroperiod (weeks)	Wood frog maximum egg mass abundance	Wood frog larval survival (%)
JSP1	Natural	26.9 (2.7)	67.7 (11.1)	19.7 (1.8)	71.3 (25.4)	7.3 (1.3)
JSP2	Created (2011)	12.2 (2.4)	4.61 (1.0)	11.7 (1.1)	5.4 (1.8)	0 (0)
JSP3	Created (2008)	24.1 (1.4)	6.22 (0.7)	30.3 (3.7)	26.0 (3.5)	1.3 (0.8)
JSP4	Created (2008)	31.4 (4.1)	52.9 (11.6)	21.4 (3.4)	51.3 (9.7)	7.1 (5.8)
JSP5	Natural	56.7 (3.4)	193 (21.2)	21.6 (1.9)	111.3 (23.6)	15.0 (4.8)

*Note:* Hydrological parameters and egg mass abundance were measured on a weekly basis from 2014 through 2023. Larval survival was estimated in 2018, 2019, and 2020. Values are means (SE).

Mean hydroperiod over the study period was 21.5 ± 1.3 weeks, but hydroperiods (range = 6–45 weeks) and dry down dates (range 25 March to 7 November) were highly variable among pools and years (Table 1). Vernal pools were generally shallow (Table 1), with a mean depth of 36.1 ± 2.3 cm (range = 4.4–81.0 cm). Mean vernal pool volume during the breeding season was 52.8 ± 9.5 m^3^ (range = 1.3–291.3 m^3^). The most notable difference among natural and created pools was related to size. Natural pools (mean depth of 48.0 ± 3.5 cm) were deeper than created pools (mean depth of 28.2 ± 2.1 cm). Other within‐pool environmental parameters, such as DO (mean = 47.6% SAT ± 2.4) and conductivity (mean = 0.05 *μ*Scm^−1^ ± 0.002) were similar among all pools. Despite the presence of roads within the wood frog breeding zone at the fragmented site (Figure 1), the overall landscape context at the two locations within the park was similar. Forest cover (range = 47.6%–68.5%) was not significantly different between the isolated and fragmented sites.

### 3.2 Within‐Population Genetic Diversity

Three samples failed to amplify in > 3 loci and were subsequently dropped. Because we found no significant between‐year differentiation in the variation of allele frequencies from 2021 and 2022, we combined the samples collected from both years.

We successfully genotyped 23–45 wood frog samples per pool for a total of 179 individuals after removing full siblings (*n* = 7 individuals dropped from full‐sibling pairs) from the analysis (Table 2). We dropped two loci (C83 and D77) because each had null allele frequencies > 20%. After dropping these loci, because the overall average percentage of null alleles was < 10%, and null alleles have been found to have minor impacts on genetic distance estimates (Chapuis and Estoup 2007), all seven other loci were retained for downstream analyses. Power simulations indicated that with these seven loci, their polymorphisms, and our sample sizes, we had high resolution for detecting population differentiation. The simulated probability of detecting *F*_*ST*_ values as low as 0.01 was 100% and the probability of detecting *F*_*ST*_ values as low as 0.005 was over 99%.

**TABLE 2:** Number of successfully genotyped individuals after removing full siblings (*N*) and genetic variation estimates from 7 microsatellite loci across in the wood frog the five sampled vernal pools in Jacobsburg State Park, PA.

	JSP1	JSP2	JSP3	JSP4	JSP5
*N*	45	23	38	36	37
*A*_*O*_	12.43	6.7	13.6	12	12.6
*AR*	10.7	6.7	11.9	10.9	11.3
*N*_*p*_	0.38	0.25	1.05	0.40	0.56
*H*_*E*_ (SE)	0.88 (0.01)	0.84 (0.01)	0.84 (0.01)	0.84 (0.01)	0.84 (0.01)
*H*_*O*_ (SE)	0.87 (0.03)	0.85 (0.06)	0.85 (0.05)	0.87 (0.03)	0.79 (0.03)
*F*_*IS*_	0.001	−0.015	0.039	0.005	0.093
*N*_*b*_ (CI)	55 (37–87)	27 (12–54)	56 (37–88)	56 (36–92)	70 (46–111)

*Note: A*_*O*_ is the mean number of alleles, *AR* is the allelic richness rarefied to 23 individuals (smallest sample size, JSP2), *N*_*p*_ is the rarefied number of private alleles, *H*_*E*_ is the mean expected heterozygosity (interlocus SE), and *H*_*O*_ is the mean observed heterozygosity (interlocus SE). *F*_*IS*_ is the inbreeding coefficient with values ranging from −1 to 1 where substantial negative values indicate an excess of heterozygotes and substantial positive values suggest inbreeding or undetected null alleles. *N*_*b*_ is the effective number of breeders at each pool (95% confidence interval).

All loci were highly polymorphic with a mean of *N* = 9.4 alleles across all loci and populations. From those seven loci included in all analyses, we did not detect scoring errors nor LD but did detect deviations from HWE in D20 and D32 in some populations. Because neither of these loci showed significant deviations across most populations, we kept them in downstream analyses.

After rarefaction, allelic richness was highest for JSP3 and lowest for JSP2 (Table 2). Similarly, levels of rarefied estimates of private alleles were highest for JSP3 and lowest for JSP2, and private alleles were generally rare, ranging from 0.25 to 1.04 alleles per pool. Observed heterozygosity was also high with a mean of *H*_*O*_ = 0.84. Pool specific observed heterozygosities ranged from 0.79 to 0.87. Additionally, pool specific *F*_*IS*_ ranged from 0 to 0.093. *F*_*IS*_ values were not significantly different from zero and therefore did not indicate sampling bias from within‐pond relatedness. The estimates for effective number of breeders calculated in COLONY (Jones and Wang 2010) using sibship frequency varied across vernal pools. All estimates had overlapping 95% confidence intervals, and JSP2 had the smallest *N*_*b*_ estimate of 27 compared to the largest estimate at JSP5 of 70. *N*_*b*_ estimates were similar for JSP1, JSP3, and JSP4 at 55, 56, and 56, respectively.

### 3.3 Population Differentiation and Structure

We found high genetic connectivity with evidence of genetic structure from our analyses. Pairwise *F*_*ST*_ values ranged from 0.008 to 0.021 (Table 3). Significance tests of pairwise *F*_*ST*_ values suggested that JSP2, the smallest created pool, was significantly differentiated from all other ponds. No other pairs were significantly differentiated.

**TABLE 3:** Genetic and geographic distances between wood frog vernal pool populations within Jacobsburg State Park, PA.

	JSP1	JSP2	JSP3	JSP4	JSP5
JSP1	—	42	87	684	876
JSP2	0.02***	—	125	651	838
JSP3	0.006	0.022***	—	771	962
JSP4	0.002	0.017***	0.003	—	241
JSP5	0.002	0.015**	0.06	0.001	—

*Note:* Pairwise *F*_*ST*_ values are reported below the diagonal, and significantly differentiated pools using a permutation test are bolded. Euclidean distances (m) between pools calculated with the haversine formula are reported above the diagonal. **p < 0.01, ***p < 0.001 after Bonferroni correction.

**FIGURE 3:**
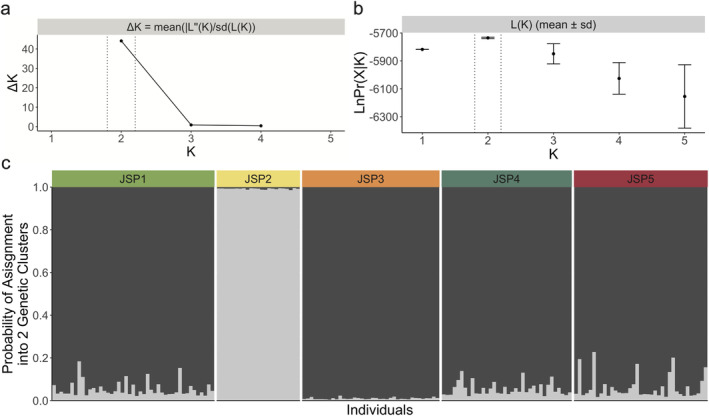
STRUCTURE analysis to estimate the number of unique genetic clusters (*K*) and the probability of assignment of each individual to a genetic cluster. (a) Pritchard, Stephens, and Donnelly 2000 and (b) Evanno, Regnaut, and Goudet 2005 methods for estimating *K*. (c) Probability of assignment of each individual (represented by single colored bar) from each vernal pool, listed in numerical order, to either of the two genetic clusters (*K* = 2). The proportion of each colored bar in dark gray indicates the probability of assignment to one genetic cluster, and the proportion of light gray indicates the probability of assignment to a second genetic cluster.

We found 2 unique genetic clusters for these data using the methods of Pritchard, Stephens, and Donnelly 2000 (Figure 3a) and Evanno, Regnaut, and Goudet 2005 (Figure 3b). Using STRUCTURE (Pritchard, Stephens, and Donnelly 2000) analyses, we identified all individuals sampled at JSP1, JSP3, JSP4, and JSP5 to have a high probability of assignment (> 0.77) to one unique genetic cluster (Figure 3c). All individuals we genotyped at JSP2 had a high cluster membership value (> 0.98) to a second unique genetic cluster (Figure 3c). These unique clusters, however, were not spatially disjunct as JSP2 is located within 100 m of both JSP1 and JSP3 (Table 3).

The AMOVA analyses suggested that neither road proximity nor created vernal pools are strongly contributing to the genetic structuring of the wetlands (Table 4). For all three models, ≥ 92% of the genetic variation was within individuals (i.e., differences between the two alleles of a diploid individual). When pools were analyzed by level of fragmentation by roads in Model 1, 0% of the total variation was explained among the isolated and fragmented pools (*P*_*Among groups*_ = 0.999). Model 2, with natural vernal pools placed in one group and constructed pools in the other, similarly explained 0% total variance among the two groups (*P*_*Among groups*_ = 0.94). Model 3, with natural vernal pools in one group and each created pool in its own group, suggested no support for differentiation among the groups. This model explained minimal total variance (0.28%), and the effect was not significant (*P*_*Among groups*_ = 0.094).

**TABLE 4:** Analysis of molecular variance (AMOVA) model analyses of wood frogs breeding in natural and created vernal pools.

Model	Variable	df	Sum of Squares	Variance of Components	% Variation	*p*
M1	Among groups	1	3.91	0	0	0.999
	Among pools within groups	3	16.42	0.03	1	**0.001** **
	Among individuals	174	578.58	0.22	7	**0.001** **
	Within individuals	179	516.5	2.89	92	**0.001** **
M2	Among groups	1	4.48	0	0	0.94
	Among pools within groups	3	15.84	0.03	0.90	**0.001** **
	Among individuals	174	578.58	0.22	7.02	**0.001** **
	Within individuals	179	516.5	2.89	92.08	**0.001** **
M3	Among groups	3	15.62	0.01	0.28	0.094
	Among pools within groups	1	4.7	0.02	0.54	**0.018** *
	Among individuals	174	578.58	0.22	7.02	**0.001** **
	Within individuals	179	516.5	2.89	92.15	**0.001** **

*Note:* Bold values indicate significant results (*p* < 0.05) using a permutation test (9999 permutations).

* *p* < 0.05.

** *p* < 0.01.

### 3.4 Gene Flow

BAYESASS (Wilson and Rannala 2003) indicated that most of the inter‐pool rates of gene flow in our analysis are indistinguishable from those generated by uninformative data. We discovered, however, one rate that fell outside of the confidence interval provided for comparison with uninformative data, representing a measurable dispersal rate given our genotypic data. BAYESASS gave an estimate of 0.0903 (0.0031–0.1775) from JSP1 to JSP3. That is, for JSP3, 9.03% of individuals sampled had parents that were inferred to have come from JSP1.

### 3.5 Bottleneck Detection

While we did conduct bottleneck analysis using the TPM, the outcomes displayed variability and were closely tied to the specific parameter configurations required for conducting analyses involving two‐phase models, such as the selection of prior values governing the degree of SMM and the variance of the geometric distribution. These data were therefore omitted.

**FIGURE 4:**
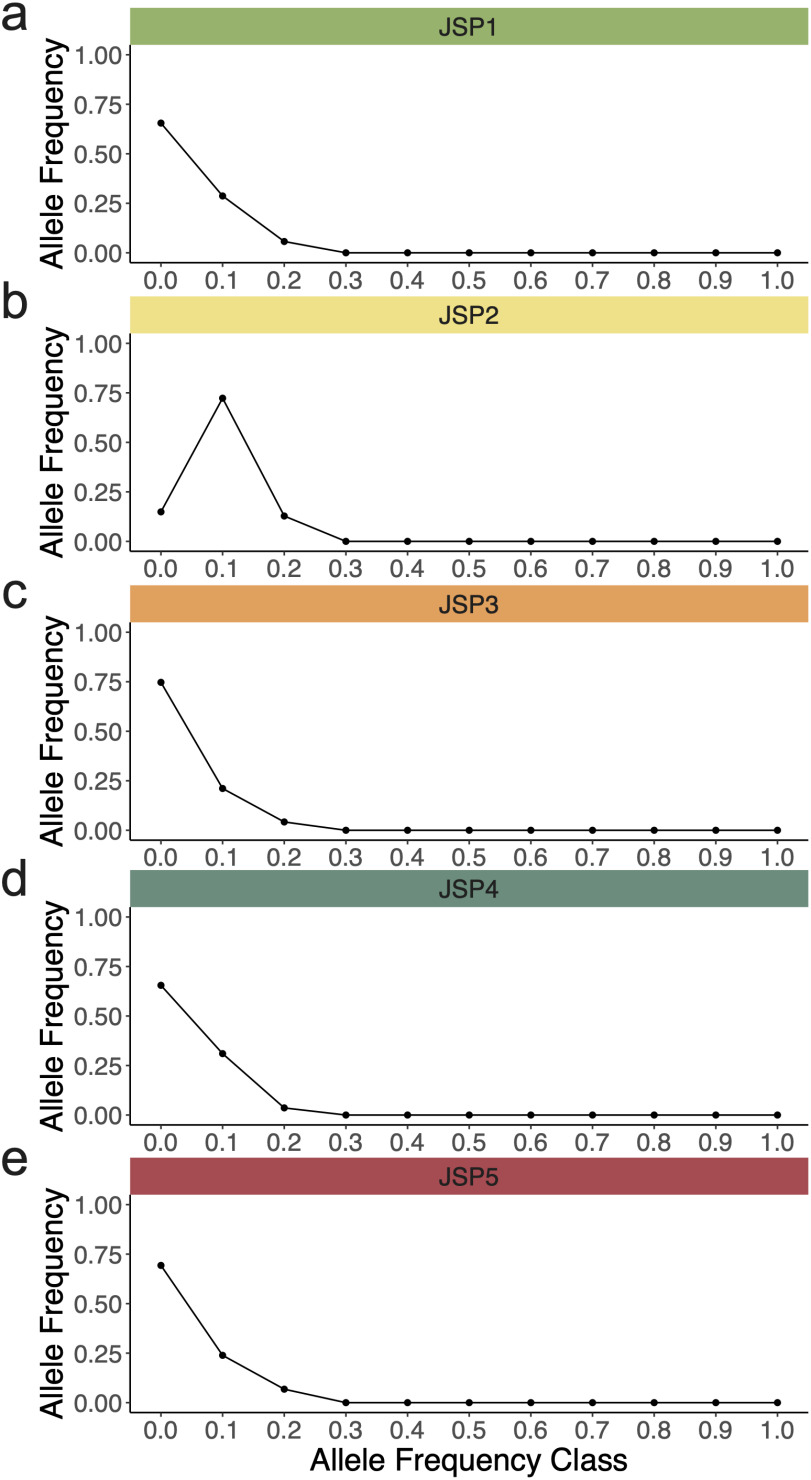
Qualitative test of mode‐shift for the detection of recent genetic bottlenecks at (a) JSP1, (b) JSP2, (c) JSP3, (d) JSP4, and (e) JSP5. L‐shaped distributions of allele frequency suggest the absence of a bottleneck whereas a characteristic mode‐shift distortion indicates a recent bottleneck has caused alleles at low frequency (i.e., rare alleles) to become less abundant than alleles in an intermediate allele frequency class (i.e., common alleles).

Under the more conservative SMM, only JSP2 (*p* < 0.01) and JSP4 (*p* < 0.05) showed a significant heterozygosity excess (Table 5). In addition, the mode‐shift indicator suggested low‐frequency alleles were present in all populations besides JSP2 (Figure 4). The L‐shaped distribution of allele frequencies in JSP1, JSP3, JSP4, and JSP5 suggested a healthy population. Finally, signatures of genetic bottlenecks were detected in JSP2 and JSP3 using the M‐ratio method (*M* < 0.68). Variations in results using these different methods reveal differential sensitivity of bottleneck detection methods (Williamson‐Natesan 2005; Peery et al. 2012), and only JSP2 was found to have undergone a genetic bottleneck event under all three tests.

**TABLE 5:** *p*‐values from a Wilcoxon heterozygosity test for the stepwise mutation model (SMM), modes obtained from the mode‐shift indicator, and calculated *M*‐ratios from five vernal pools within Jacobsburg State Park, PA.

Pool	SMM	Mode‐shift test	*M*‐ratio
JSP1	0.109	Normal L‐Shaped	0.848
JSP2	**0.008** **	**Shifted Mode**	**0.585**
JSP3	0.938	Normal L‐Shaped	**0.545**
JSP4	**0.039** *	Normal L‐Shaped	0.724
JSP5	0.578	Normal L‐Shaped	0.680

*Note:* Bold values indicate: (1) significant p values (*p* < 0.05) for the SMM, (2) Shifted Mode, suggesting a bottleneck, and (3) M‐ratio values less than 0.68, which are indicative of a population bottleneck.

* *p* < 0.05.

** *p* < 0.01.

## 4 Discussion

The initial goal of this long‐term monitoring study was to compare environmental parameters and reproductive success of indicator species between natural and created pools in the same area. Previous evaluations of vernal pool restoration attempts have yielded conflicting results. Some studies indicate that created pools rarely replace the function lost in the destruction of natural pools (Lichko and Calhoun 2003; Calhoun et al. 2014), while others demonstrate that created vernals pools can match the value and function of nearby natural pools (Brown et al. 2012; Rothenberger et al. 2019). Either way, restoration practitioners generally agree that created pools can only be reliably evaluated through long‐term monitoring of environmental factors known to influence reproductive success and persistence of indicator species populations.

Monitoring of environmental variables yielded some key implications for vernal pool restoration practice. Vernal pool hydroperiod, volume, depth, and forest land cover within the life zone are more important predictors of indicator species reproductive success and larval survival than pool type (i.e., natural or created). While overall larval survival of the wood frog and spotted salamander were significantly higher in natural than created pools in Jacobsburg State Park, these indicators of restoration success were comparable at one created pool (JSP4) in this study. Long‐term monitoring of natural, created, and restored pools at other locations in Pennsylvania and New Jersey has demonstrated that some created pools can exceed the functional success of nearby natural pools when they are well‐established (i.e., > 10 years old) and designed with key habitat criteria in mind (Rothenberger et al. 2019; Rothenberger and Baranovic 2020). Also, vernal pool restoration projects should be accompanied by extensive monitoring studies that include measures beyond amphibian egg mass counts. For example, in our study, JSP3 had a mean total egg mass abundance of 26 (i.e., both indicator species combined) but a mean overall larval survival of 1.3%, and JSP2 had a mean total egg mass abundance of 6 and mean overall larval survival of 0%. Vernal pools with wood frog and spotted salamander egg masses do not always have high overall larval survival rates.

The next goal of this study was to use population genetics tools to evaluate genetic diversity within and between pools, population structuring, and gene flow for wood frogs. This was an important next step because within‐pool measures of indicator species abundance do not yield direct information on genetic diversity and dispersal ability, which are crucial to a population's evolutionary flexibility and capacity to adapt to changing landscapes. Two of the created pools in our study (JSP3 and JSP4) had comparable measurements of allelic richness and heterozygosity to the natural pools and were included in the same genetic cluster as the natural pools. Our results reinforce the idea that created vernal pools can support populations with similar genetic diversity to those found in natural reference pools. Similarly, Millikin et al. (2023) found spotted salamander genetic diversity levels in created pools ≤ 5 years old as high as nearby natural pools.

Most population genetic studies on wood frogs report little to no evidence of genetic structuring or differentiation between breeding pools located up to 50 km apart (e.g., Lee‐Yaw et al. 2009; Richardson 2012; Coster, Babbitt, and Kovach 2015). Adult wood frogs breed exclusively at the same pool, but some juveniles disperse, and these individuals connect neighboring pools (Berven and Grudzien 1990). In this study, however, we found the created pool JSP2 to be genetically differentiated with a much lower rarefied allelic richness (*AR* = 6.7) than the other four pools (average *AR* = 11.2 ± 0.26). This differentiation is unexpected given JSP2's close proximity (< 1 km) to other natural and created pools. The main environmental parameters that set JSP2 apart are its hydroperiod (11.7 ± 1.1 weeks), shallow depth (12.2 ± 2.4 cm), and small water volume (4.61 ± 1.0 m^3^). Wood frogs generally emerge from vernal pools as metamorphs in July or August in Pennsylvania, but our data show that JSP2 was always dry by June. This early drying led to mortality of colonizers. JSP2 shows the signature of a genetic bottleneck across all three bottleneck detection tests (i.e., SMM heterozygosity test, mode‐shift indicator, and *M*‐ratio test), and an effective number of breeders that is less than half of that at each of the other pools. Together, these findings point to this created pool as being a population sink with low‐quality habitat where individuals attempt to breed but fail to survive, leading to increased genetic drift and reduced genetic diversity.

Because wood frogs spend most of their lives foraging and resting in terrestrial habitat surrounding vernal pools, conservation of the upland matrix is as integral as the pool itself (Calhoun et al. 2017). Adult amphibians will commonly use pools as stepping stones when moving to summer and winter refugia (Semlitsch 1998), and our data suggest that the pools within the park appear to be connected by dispersal events. JSP1, a natural pool with high functional success, is likely a population source for neighboring pools. Estimates of migration rates between pools with BAYESASS reveal recent gene flow from JSP1 to JSP3. This result is supported by previous research of wood frog movement in the terrestrial habitat surrounding these pools. Engberg and Rothenberger (2015) investigated the effect of road proximity on upland movement patterns of wood frogs using drift fences and pitfall traps around pools at both the isolated location and the location fragmented by roads. At the isolated site where there were no barriers to movement within the surrounding 1000 m, wood frogs were uniformly abundant in traps around the pools. At the other location in the park, however, wood frogs were trapped at a lower frequency near roads than expected by chance. Although this may indicate that the presence of roads may reduce the amount of upland habitat wood frogs use, our AMOVA models did not find significant genetic variation across the natural and created pools or pools closer to roads. The roads in this study do not entirely hinder their movement, suggesting that forest fragmentation has not reached a critical threshold. Instead, small differences in wood frog population structure at a fine‐scale level have been linked to extensive road density and highway networks (Crosby, Licht, and Fu 2009).

The restoration implications drawn from the case study of JSP2 and its local challenge in conservation genetics being a population sink despite having egg masses underscore the importance of ongoing monitoring efforts and strategic adaptive management strategies. Adaptive management, in this context, refers to an iterative process of decision‐making that integrates scientific understanding with management actions (Schlatter, Faist, and Collinge 2016). This allows for flexibility and adjustment of actions in response to changing conditions or new information. Our findings underscore the importance of considering multiple factors, including hydrology, climate change, the upland habitat, and genetic diversity, in vernal pool restoration planning and management.

Managing hydroperiods at the pool level is vital for the success of any vernal pool creation or restoration project. Improper hydrological regimes are a primary reason for vernal pool failure (Gamble and Mitsch 2009; Korfel et al. 2010; Denton and Richter 2013). Restoration practitioners can manipulate hydroperiods by including a synthetic liner (e.g., JSP4), adjusting pool volume, or constructing pools in certain soil types (Calhoun et al. 2014). Soil compaction in created pools may influence hydroperiod (Whittecar and Daniels 1999; Gamble and Mitsch 2009), and if the soils have sufficient clay, they can be readily compacted to hold water (Biebighauser 2003, 2011). Vernal pool hydroperiods are also highly sensitive to changes in temperature and precipitation patterns (Calhoun et al. 2017). For example, JSP4 and JSP5 were initially classified as short‐cycle pools (12–20 weeks; Rothenberger et al. 2019, Rothenberger and Baranovic 2020), but these pools have gradually shifted into the long‐cycle category (20–35 weeks) as local precipitation totals increase above the historical (100‐year) average. Responses to climate change will vary across regions, but in the Northeastern United States, extreme precipitation has increased rapidly over the past century (Huang et al. 2021). Longer hydroperiods are known to increase numbers of amphibian species and invertebrates that prey upon target vernal pool indicators (Babbitt, Baber, and Tarr 2003; Calhoun et al. 2014; Rothenberger and Baranovic 2020). Therefore, managing hydroperiods to promote indicator species establishment and continued recruitment is important for avoiding persistent population sinks and promoting indicator species' capacity to adapt to this change.

The upland habitat surrounding the vernal pool that encompasses the indicator species' life zone is also important to consider within the management framework. As land conversion and urbanization continue, it is particularly valuable to continue to create and restore vernal pools at fragmented sites where former breeding habitat has become inaccessible because of man‐made barriers, and the number of breeding wetlands is too small to support resilient amphibian populations (Stokes et al. 2021). This study reinforces the idea that it is especially important to create and restore vernal pools that are paired with the conservation of existing pools to promote colonization and between‐pool flow of genetic material (Vasconcelos and Calhoun 2006; Calhoun et al. 2014; Rothenberger et al. 2019).

Genetic monitoring, coupled with environmental assessments, offers valuable insights into population dynamics and informs adaptive management strategies (Schwartz, Luikart, and Waples 2007; Stephens, Tolley, and Da Silva 2022). For instance, identifying population sinks like JSP2 suggests targeted interventions to enhance habitat quality and promote population persistence, such as adding synthetic liners or adjusting physical characteristics. Our work establishes a baseline for future monitoring aimed at tracking long‐term trends for conservation purposes.

While our study provides valuable insights for these 5 vernal pools, it may not fully represent the genetic diversity across all vernal pools within the park or the broader landscape. We acknowledge that future studies with larger sample sizes and extended geographic coverage could help validate and expand upon our findings. That being said, by corroborating our ecological observations with genetic evidence, we not only strengthen the validity of our conclusions but also demonstrate the robustness of our research methodology. This is important and novel because so few vernal pool studies actually integrate environmental and genetic monitoring. To address this limitation, landscape genetics offers a promising approach (e.g., Coster, Babbitt, and Kovach 2015; Coster et al. 2015; Homola, Loftin, and Kinnison 2019). This field integrates genetic data with landscape features to understand their influence on gene flow and population dynamics. Incorporating landscape genetics into future studies can provide a more comprehensive understanding of how habitat fragmentation and landscape heterogeneity affect amphibian populations, thereby informing more effective conservation strategies for the broader landscape context.

The implications of our results at the local scale extend beyond our study area. Amphibians and wetlands face threats globally (Nolan et al. 2023), and restoration attempts play a crucial role in conservation efforts. However, the success of these restoration endeavors relies on more than the implementation of just any action alone—it requires clear goals and robust monitoring programs to evaluate progress and adapt management strategies as needed. As we move forward, it is imperative that we recognize the value of holistic approaches to these ecosystems' conservation. By understanding the broader context and collaborating across disciplines, we can work toward the conservation of amphibian populations and their habitats on a global scale. Ultimately, the success of conservation efforts relies on our ability to learn from local studies and apply these lessons to inform comprehensive and effective conservation practices worldwide.

## Author Contributions

**Declan M. Winters:** conceptualization (equal), data curation (equal), formal analysis (lead), investigation (lead), methodology (equal), writing – original draft (lead), writing – review and editing (equal). **Emily Wilson:** conceptualization (equal), data curation (equal), formal analysis (equal), investigation (equal), methodology (equal), writing – original draft (supporting), writing – review and editing (equal). **Stephanie S. Coster:** formal analysis (supporting), methodology (supporting), writing – review and editing (equal). **Megan B. Rothenberger:** conceptualization (equal), data curation (equal), formal analysis (equal), investigation (equal), methodology (equal), supervision (lead), writing – original draft (supporting), writing – review and editing (lead).

## Conflicts of Interest

The authors declare no conflicts of interest.

## Supporting information


Appendix S1.

## Data Availability

Microsatellite genotypes and ordination matrices are archived in the online Supporting Information.

## Appendix 1 Details of sample collection

### 1.1

In 2022, DNA was extracted from about 20 wood frog eggs at each vernal pool in Jacobsburg State Park, PA. Eggs were sampled from different egg masses, and a new pair of gloves were worn between each collection to prevent contamination. Tubes were filled with 0.5 mL 95% ethanol and placed on ice in the field. Two eggs were taken from a restored vernal pool at The Lee and Virginia Graver Arboretum in Bushkill Township, PA to develop a positive control.

To increase the power of our dataset and sample more individuals at each pool, we performed a toe‐clipping procedure on adult wood frogs in 2023 in addition to collecting more eggs. Adults were collected using aquatic funnel traps illuminated with glow sticks (Antonishak, Muñoz, and Miller 2017). Prior to deployment, all field equipments were scrubbed with a 2% bleach solution and dried for 48 h to prevent the spread of *Ranavirus*, *Batrachochytrium dendrobatidis* (the amphibian chytrid fungus), and other pathogens (Johnson and Speare 2003; Bryan et al. 2009). Two variations (i.e., “Minnow” and “Crawfish”) of unaltered aquatic funnel traps (Frabill, Plano Molding Company, Plano, Illinois, USA) were deployed to maximize capture rates. Both are torpedo shaped, constructed of black vinyl coated steel with mesh, and have cone‐shaped openings (Figure A1a). This allows amphibians to enter with a limited chance of escape. Waterproof foam floats (FOAMULAR, Owens Corning, Toledo, Ohio, USA) were constructed and placed inside each trap to raise the entire unit partially out of the water to prevent the drowning of any air‐breathing animals that may enter (Figure A1b). Traps were deployed every 10 m along the perimeter of the pool and attached to the bank using rope and a wooden stake to prevent drifting. Openings were oriented perpendicular to the pool's edge in deep enough water so that everything but the float fully submerged in water (typically about 1–2 m from the bank). A total of 42 traps were deployed across all five vernal pools, resulting in 4–13 traps per pool depending on size, and trap type was alternated whenever possible (i.e., two traps of the same type were placed beside each other in pools with an odd number). An activated green glow stick was hung inside each trap (HSG US LLC, Brooklyn, New York, USA) (Figure A1c). Amphibian movements toward vernal pools upon emergence from hibernation mostly occur at night (Todd and Winne 2006), and glow sticks illuminated at night can increase funnel trap capture rates for adult vernal pool amphibians including the wood frog (Antonishak, Muñoz, and Miller 2017). Glow sticks were therefore activated between 1600 h and 2000 h, allowing them to reach a maximum brightness during the nighttime before traps were checked the next day and each glow stick replaced. Traps were only ever illuminated with a new glow stick and placed in the pool's water during oviposition.

To collect the tissue sample, one frog was handled at a time, and a fresh pair of gloves were worn between each procedure. The frog rested on the fingers while the thumb held it gently in place. A slight pressure was applied to keep the frog from escaping. Sharp, scientific grade scissors were used to take 5–10 mm of tissue from the fourth toe from the inside of the right, posterior leg (Newman and Squire 2001; Crosby, Licht, and Fu 2009). Tweezers were used to place samples into a microcentrifuge collection tube (one individual per tube), and samples were topped with 95% ethanol. Collections were placed on ice in a small cooler in the field. After each toe was clipped, all instruments were soaked with a 3% hydrogen peroxide solution for 10 s. The hydrogen peroxide was frequently changed as it loses its potency with repeated uses. Instruments were then rinsed with DI water before clipping the next individual. The spread of pathogenic organisms, such as the frog chytrid fungus, may occur because of toe‐clipping procedures. This can be minimized by strictly following these procedures to disinfect instruments between frogs and using a new pair of gloves for each new procedure (Berger et al. 1998). Furthermore, open wounds were sealed with Vetbond, a cyanoacrylate compound, to reduce the likelihood of entry of pathogens. The National Parks & Wildlife Service further recommends the application of a 1% povidone‐iodine disinfectant after any surgical procedure (National Parks and Wildlife Service 2000). A small amount was used after the wound sealant before the frog was rinsed with spring water and released back into the pool it was originally captured in.

## Appendix 2 Multiplex design and primer concentrations for PCR

**TABLE A1:** Characteristics of 9 wood frog microsatellite DNA loci and fluorescent‐labeled tails for primer and multiplex designs.

Name	Repeat motif	Fluorphore/Forward tail	Multiplex ID	Primer sequence (5′‐3′)	Size range (bp)
C11	(TACA)_9_	6‐FAM	B	F: 5′‐GCCTCCCTCGCGCCATTACTTTCAGTTTCAAAAGGCAG‐3′	105–185
				R: 5′‐TACACAGTGCTTCACAAGTTCC‐3′	
C41	(TACA)_8_	NED	B	F: 5′‐CAGGACCAGGCTACCGTGGTCAAAACACAGATGCACAATC‐3′	80–135
				R: 5′‐ACAAAACAGGAATCGGTCATAC‐3′	
C52	(TACA)_17_	HEX	A	F: 5′‐GCCTTGCCAGCCCGCCCATACAACCGTGATTACAAAAG‐3′	125–210
				R: 5′‐ATATACCACCCTTCCAGAGATG‐3′	
C63	(TACA)_12_	HEX	B	F: 5′‐GCCTTGCCAGCCCGCCAGAAAATTGCCGAAAAGG‐3′	125–255
				R: 5′‐TGGGCTTAAGAAACAAAAGAAC‐3′	
C83	(TACA)_10_	NED	A	F: 5′‐CAGGACCAGGCTACCGTGTGCATTGTTTTACTTATGTGTGAAG‐3′	95–135
				R: 5′‐TTACCTGGTCTTGTAATGGGAC‐3′	
D20	(TAGA)_18_	6‐FAM	C	F: 5′‐GCCTCCCTCGCGCCAGTTACTGTGGAGGTGATGTCTG‐3′	200–280
				R: 5′‐TTCTATATCAAGCACCCATCTG‐3′	
D32	(TAGA)_11_	NED	A	F: 5′‐CAGGACCAGGCTACCGTGGGACACACAATTCCTTGGTTC‐3′	160–230
				R: 5′‐GAGGAGATTTCCAAAACAATCC‐3′	
D55	(TAGA)_25_	6‐FAM	A	F: 5′‐GCCTCCCTCGCGCCAGAGTTGGGACTCCTGAATAGAG‐3′	150–250
				R: 5′‐AGTCTTTGCTTTGTAAATTGGC‐3′	
D77	(TAGA)_15_	NED	C	F: 5′‐CAGGACCAGGCTACCGTGGCATAGTGACATGTTTCACCC‐3′	165–225
				R: 5′‐GTAAGAGAAGGTTCACCTCAGC‐3′	
Tail A	—	6‐FAM	—	5′‐GCCTCCCTCGCGCCA‐3′	—
Tail B	—	HEX	—	5′‐GCCTTGCCAGCCCGC‐3′	—
Tail C	—	NED	—	5′‐CAGGACCAGGCTACCGTG‐3′	—

**TABLE A2:** Primer final concentrations used in PCR reactions.

Name	Tailed‐forward primer (μM)	Reverse primer (μM)	Fluorescent tail (μM)
C11	0.1	0.2	0.2
C41	0.1	0.2	0.2
C52	0.1	0.2	0.2
C63	0.1	0.2	0.2
C83	0.15	0.3	0.3
D20	0.1	0.2	0.2
D32	0.1	0.2	0.2
D55	0.1	0.2	0.2
D77	0.1	0.2	0.2
